# Anti-*Trypanosoma cruzi* antibody profiling in patients with Chagas disease treated with benznidazole assessed by genome phage display

**DOI:** 10.1371/journal.pntd.0011019

**Published:** 2023-01-06

**Authors:** Luis Antonio Rodriguez Carnero, Andréia Kuramoto, Léa Campos de Oliveira, Jhonatas Sirino Monteiro, João Carlos Setubal, Edécio Cunha-Neto, Ester Cerdeira Sabino, Ricardo José Giordano

**Affiliations:** 1 Department of Biochemistry, Institute of Chemistry, University of São Paulo, São Paulo, SP, Brazil; 2 Heart Institute (InCor) and Division of Clinical Immunology and Allergy, University of São Paulo School of Medicine, São Paulo, SP, Brazil; 3 Department of Infeccious Diseases and Parasitology, University of São Paulo School of Medicine and Institute of Tropical Medicine, University of São Paulo School of Medicine, São Paulo, SP, Brazil; 4 Institute for Investigation in Immunology (iii), INCT, São Paulo, SP, Brazil; Universidade Federal de Minas Gerais, BRAZIL

## Abstract

**Background:**

There have been significant improvements in Chagas disease therapy and it is now widely accepted that most patients with chronic disease might benefit from therapy. However, there are challenges to monitor drug efficacy and cure for these patients, which are important impediments for current and future therapies. *Trypanosoma cruzi*-PCR is highly variable while IgG seroconversion takes decades yielding variable results depending on the antigen(s) used for the assay.

**Methods and results:**

We used the genomic phage display (gPhage) platform to perform a pairwise comparison of antigens and epitopes recognized by twenty individual patients with chronic Chagas disease before and after treatment with benznidazole. In total, we mapped 54,473 *T*. *cruzi* epitopes recognized by IgG from individual patients (N = 20) before benznidazole treatment. After treatment, the number of epitopes recognized by all patients was significantly smaller (21,254), a reduction consistent with a decrease in anti-*T*. *cruzi* antibodies. Most of these epitopes represent distinct fragments from the same protein and could, therefore, be grouped into 80 clusters of antigens. After three years of treatment with benznidazole, we observed a 64% reduction in the number of clusters of antigens recognized by patients (59 clusters before versus 21 clusters after treatment). The most abundant antigenic clusters recognized by patients correspond to the surface antigen CA-2 (B13) followed by the microtubule associated antigen, which highlights the value of these epitopes in Chagas disease diagnosis. Most importantly, quantitative pairwise comparison of gPhage data allowed for the prediction of patient response to treatment based on PCR status.

**Principal finding:**

Here, we compiled a list of antigens and epitopes preferentially recognized by Chagas disease patients before and after benznidazole treatment. Next, we observed that gPhage data correlated with patient PCR-status and could, therefore, predict patient response to treatment. Moreover, gPhage results suggest that overall, independent of PCR status, treatment led to a reduction in the presence of *T*. *cruzi*-specific antibody levels and the number of antigens and epitopes recognized by these patients.

**Conclusion:**

The gPhage platform use of unbiased library of antigens, which is different from conventional serological assays that rely on predetermined antigens, is a contribution for the development of novel diagnostic tools for Chagas disease.

## Author summary

Chagas disease, caused by the single-celled parasite *Trypanosoma cruzi*, can be a life-treating and debilitating illness. Because there is no vaccine and currently the only two available drugs are most effective if used during the early acute stage of the disease, treatment options for infected individuals are limited. Most individuals will only find out they have Chagas disease during a routine medical examination or in blood bank while donating blood, in which cases, they are already chronically infected. At this stage, treatment will not undo clinical manifestations (i.e., cardiomyopathy) but may eliminate the parasite and prevent disease progression. Currently, polymerase chain reaction (PCR) and serological assays are the only diagnostic tools available, both with limitations in sensitivity and accuracy. The lack of effective molecular markers thus prevents physicians to determine whether a patient is parasite free and cured from the disease. It also has important implications for the development of new drugs to treat Chagas disease. Here, we studied the reactivity of anti-*T*. *cruzi* antibodies in sera from a cohort of 20 patients that underwent treatment for Chagas disease using a new method developed by our group named gPhage. Using gPhage, we scanned all *T*. *cruzi* proteins to identify those that were reactive with the antibodies from each individual patient before and after treatment. In sum, gPhage data correlated with patient PCR-status and could, therefore, predict patient response to treatment. It also revealed a new set of *T*. *cruzi* proteins that could be useful for the development of future diagnostic methods.

## Introduction

Caused by the protozoan parasite *Trypanosoma cruzi*, Chagas disease is a neglected tropical disease that affects an estimated 6 to 8 million people worldwide, resulting in approximately 50,000 annual deaths [1]. In endemic areas, *T*. *cruzi* is mostly transmitted to human hosts by infected feces of triatomine insect vectors [2]. In non-endemic regions, other forms of transmission may still occur, such as congenital, transfusion, organ transplant or by ingesting contaminated food. Neither vaccine nor a fully-effective treatment is available for Chagas disease [2,3].

The challenge to treat Chagas disease is in part due to the nature of the disease and its two distinct phases: acute and chronic. Upon infection, the individual enters the acute phase of the disease with high blood parasitemia but usually without specific symptoms. Therefore, it often goes unnoticed and the great majority of patients is not diagnosed until later in life when they are already with chronic Chagas disease [2,4]. The chronic phase is often asymptomatic and the majority of infected people will eventually die of natural causes or other illnesses. Only a fraction of them (approximately 30%) will develop, over the course of decades, the characteristic heart or gastrointestinal Chagas disease (estimated at 9.2 events/1,000 infected person-years) [5].

The two available drugs for Chagas disease therapy are benznidazole and nifurtimox. Treatment with these drugs has shown a better performance when applied at the early stages of the disease (acute phase) [2,6]. Unfortunately, this is seldom the case and most people will only realize they have the disease much later in life, such as during a routine visit to their doctor or by screening in blood banks. Currently, serological and polymerase chain reaction (PCR) methods are used to determine presence and parasite load [7]. Nevertheless, due to the very low parasitemia during the chronic stage of the disease, even the very sensitive PCR method is not ideal, making it difficult, for instance, to assess drug efficacy. ELISA and immunofluoresce assays are the most common methods to diagnose chronic Chagas disease, with certain limitations for current available tests [8], but seronegativation may take decades [2]. So, to date, there are no ideal molecular markers or methods to determine disease status and cure.

Currently, benznidazole is the main treatment agent as it has shown to be less toxic [9] and possibly more effective in reducing parasite load [6]. Nevertheless, it cannot revert loss of cardiac function in patients that have already developed Chagas cardiomyopathy [9,10]. Hence, the question remains whether or not parasite clearance detected by PCR is enough to establish responsiveness to treatment. Considering that there is good evidence that the presence of *T*. *cruzi*-specific antibody levels is inversely correlated with the active infection (determined by PCR) in untreated patients [11,12], the humoral response to the parasite and the epitopes recognized by these antibodies may be important indicators of disease status.

To address these questions, we employed our recently developed genomic phage display (gPhage) platform for antibody antigen identification and epitope mapping [13,14] to evaluate the antibody response of Chagas patients that underwent benznidazole treatment. The gPhage platform relies on an unbiased library of (all) *T*. *cruzi* antigens displayed on the surface of filamentous phage, which are then presented to the IgG of patients with Chagas disease to identify antigens and epitopes reactive with sera antibody. Using gPhage display, we have identified immunodominant epitopes recognized by Chagas patients before and after antitrypanosomal treatment, and observed that the reduction of reactivity to these markers could be associated with benznidazole treatment efficacy.

## Material and methods

### Ethics statement

All participants in the study provided written informed consent before enrolment and the study was approved by the Ethics Committee at School of Medicine, University of São Paulo (approval 042/12).

### Patient selection

For the gPhage screening, we selected 20 patients that underwent a full benznidazole treatment course. Sera from each patient were obtained at the beginning of the treatment and 3 years after baseline. PCR tests were performed before treatment, at baseline, and at 60 days, 6 months, 1 and 3 years after treatment. Data from PCR tests were used to classify patients as responder or non-responder to treatment according to two distinct criteria (for details, see Patient cohort in the Results and Discussion section). For validation studies by ELISA, sera from an additional cohort of 21 patients were used in addition to sera from our initial cohort of 20 patients (**S1 Table**).

### Bacterial strains

*Escherichia coli* TG1 strain was used for library production/amplification and phage display screening with patients.

### gPhage library amplification and titration

DNA encoding the gPhage library [13] was transformed into electrocompetent *E*. *coli* bacteria (TG1 strain, Lucigen) and growth in LB media until reaching *Log* phase (OD_600nm_ 0.4–0.8). *Log* phase bacteria were infected with M13KO7 helper phage (New England Biolabs) and cultured overnight (ON) at 37°C (250 rpm) in LB media containing kanamycin and ampicillin. Next day, phage particles were purified using the PEG/NaCl method [13,15]. Aliquots of the amplified phages were then kept frozen (-20°C) until use. TG1 strain bacteria in *Log* phase was infected with serial dilutions of the amplified gPhage library and cultured ON in LB agar plates containing ampicillin, which was used to calculate the library titter by colony count and expressed as Transducing Units (TU).

### Adjustment of protein concentration in sera

Protein concentration in sera was quantified by Nanodrop (280 nm)(Thermo-Fisher Scientific) and adjusted to a 60 μg/mL concentration with Phosphate Buffered Saline (PBS). Aliquots of diluted sera were frozen at -20°C until use.

### gPhage biopanning

Anti-human IgG specific for the Fc fragment (anti-Fc) (Jackson Immuno Research 1009-001-008) was immobilized on microtiter plates wells (96 microwells) (ON at 4°C), washed four times with PBS and blocked with PBS containing 2.5% bovine serum albumin (PBS/BSA) for 2h at room temperature (RT). Well coated with anti-Fc IgG were then used to capture IgG from individual patient’s sera. To perform the two-tier biopanning, 50 μL of the pre-clearing sera (T0 or T3) were added to the anti-Fc coated-wells, incubated for 2h at room temperature and the wells washed 4 times with PBS. The gPhage library (or pool of phage from the previous round of selection) was then added to each well containg the captured IgG at a concentration of 10^10^ TU in 50 μL and incubated for 2h at RT. After the incubation period, unbound phage was recovered and transferred to a new well coated with anti-Fc and pre-incubated with the sera from the same patient at the second time-point (T3 or T0). After 2 hours of incubation at RT, wells were washed 10 times with 200 μL of PBS 0.5% Tween-20 (PBST) and phage bound to the target IgG was recovered by bacterial infection at 37°C for 30 min (50 μL of *E*. *coli* TG1 strain in *log* phase). Infected bacteria were diluted in 2xTY media containing ampicillin (50 μg/mL) and co-infected with helper phage M13KO7 (10^10^ TU). Kanamycin (50 μg/mL) was then added to the cell cultures and bacteria and phage cultured ON at 37°C (250 rpm, revolutions per minute). On the following day, bacteria were centrifugated at 8,000g for 10 min and phage particles recovered from the supernatant by the PEG/NaCl method [15]. Phages were tittered and 5x10^9^ TU of phage per well were used for next rounds of selection. In total, four rounds of selection were performed. After selection, the pool of phage particles was used for large-scale sequencing. Between the second and fourth rounds of selection, aliquots of the bacteria infected with bound phages were separated for quantification of phage recovery by colony count (quantification of bound phages enrichment).

### Large-scale DNA sequencing

Large-scale DNA sequencing was performed using MiSeq Reagent Kit v2 (500 cycles) on an Illumina MiSeq equipment at the Center for Advanced Technologies in Genomics at Institute of Chemistry, University of São Paulo. Sequencing was performed as previously described [13,16]. In brief, specific oligonucleotide primers were used to amplify by PCR the gene VIII region encoding the *T*. *cruzi* antigens from phage isolated from each patient. The phage population to be sequenced was chosen according to the enrichment data in third or fourth rounds of selection (highest enrichment). The indexation was performed in two steps: one initial PCR to add the barcodes and a second using the PCR Nextera kit to complete the process. We used four different forward and reverse primers containing zero to three degenerated bases to add the diversity necessary for amplicon sequencing with the Illumina platform. Phages from third or fourth rounds were adjusted to 10^8^ TU/μL and used as template in 25 μL PCR reactions with Kappa HiFi Polymerase (Kapa Biosystems, Roche) for 20 cycles (melting: 98°C for 20 s; annealing: 65°C for 15 s; extension: 72°C x 20s). PCR products were then purified with QIAGEN PCR purification kit and quantified in Nanodrop (260 nm). An 8 cycles PCR was performed to add the index adaptors (barcodes) with the Nextera XT kit (Illumina) (melting: 98°C for 20s; annealing: 55°C for 15s; extension 72°C for 30s). The final PCR products were purified with QIAGEN PCR purification kit and quantified by PicoGreen (Thermo-Fisher Scientific). The size of the indexed PCR products was evaluated by estimation of PCR migration in ImageJ and adjusted to 4 nM concentration. Equal volumes of the adjusted concentration products were mixed and the 4 nM resulting library quantified by qPCR using a KAPA Library quantification kit (Kapa Biosystems, Roche). The library was denatured (0.2M NaOH, 95°C for 5 min) and sequenced with a MiSeq Reagent Kit v2 (500 cycles) on an Illumina MiSeq equipment.

### Bioinformatic analysis

Bioinformatic analysis was performed as reported with a few modifications [13]. Pair assembling was performed with PEAR [17] and DNA sequences extracted and sorted using the barcode sequence. Singletons were discarded and remaining sequences aligned to four *T*. *cruzi* genomes [CL Brener (GenBank: GCA_000209065.1), DM28c (GenBank: GCA_003177105.1), marinkellei (GenBank: GCA_000300495.1), Sylvio X10 (GenBank: GCA_000188675.2)] using BLAST-like alignment tool (BLAT) [18]. Inserts with less than 95% identity were discarded as well as those containing stop codons or not in the correct frame to produce a pVIII-fused peptide. Remaining inserts were translated and the resulting peptides aligned to the proteome (derived from the same *T*. *cruzi* genomes above) with BlastP [19]. Peptides with at least 60% identity with *T*. *cruzi* proteins were considered for next steps. In order to cluster the epitopes, identified peptides were sorted by abundance (decreasing order) and the first peptide (seed) removed from the list and compared with the remaining peptides using the FuzzyWuzzy package [13]. Peptides with at least 80% sequences similarity with the seed were removed from the list and included in the growing cluster. The pipeline was performed for each patient individually until all peptides were clustered. The cluster consensus sequences were submitted to BlastP to identify the related *T*. *cruzi* antigen.

### Enzyme-Linked Immunosorbent Assay (ELISA)

Peptides (B13:FGQAAAGDKPPLFGQAAAGDKPSL; MAP:YKRALPQEEEEDVGPRHVDPDHFRSTT; Hypothetical protein TcG_12368:GGFGSATTTSTPAAGGFGS-AAHTSTPAVG; R27-2: KVAEAEKQRAAEATKVAEAEKQRAA; Putative Trans-sialidase RNE97461.1: YIDGKSLGEEEVPLTGEKPLELF) were synthetized (Chinese Peptide Company) and used for the ELISA tests. Peptides were diluted to a 50 μg/mL concentration in 50 mM carbonate buffer, pH 9.0. Then, 50 μL of diluted peptides were immobilized ON in 96-well microtiter plates. Next day, the wells were washed 3 times with PBST, blocked with PBS/BSA for 2h at RT. Patient sera at 1/200 dilutions were added to the wells and incubated with the peptides for 1h at 37°C. Wells were washed 5 times with PBST, 50 μL of secondary anti-Human IgG antibody conjugated to horseradish peroxidase (Sigma, A6029) 1/5000 dilution added and the wells incubated at 37°C for 1h. ELISA were quantified using the OPD substrate (SigmaFast OPD, P9187). The reaction was stopped using 50 μL of H_2_SO_4_ per well and the absorbance read at 420 nm. We used sera from 12 healthy donors and 6 Leishmaniasis patients as negative controls for ELISA. Cut-off values of O.D._420nm_ were determined by comparison with control samples (B13: 0.11; MAP: 0.06; Hyp TcG_12368: 0.06; R27-2: 0.07; TS RNE 97461.1: 0.07).

### Statistical analysis

Software GraphPad Prism7 was used for graphical and statistical analyses. Data with normal distribution (D’Agostino-Pearson or Kolmogorov-Smirnov test) were analyzed by paired Student’s T-test. Data that did not show a normal distribution were analyzed by Wilcoxon signed-rank test.

## Results and discussion

### Patient cohort

**Fig 1:**
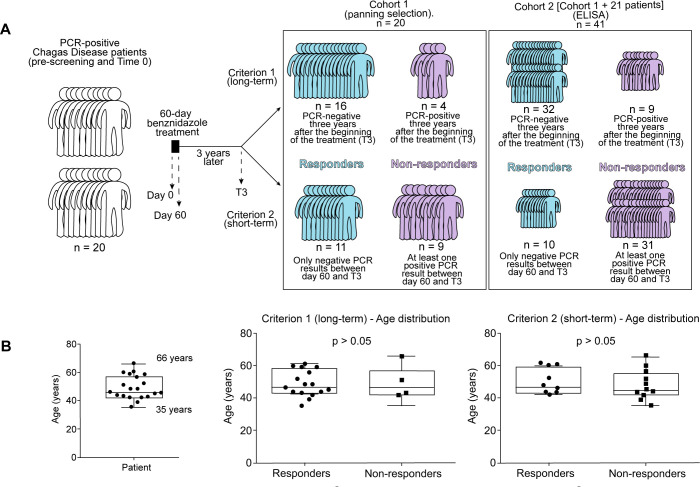
*Patient cohort profile*. (**A**) Scheme to illustrate the cohort of patients used for the gPhage selection, including an additional cohort of 21 patients used for ELISA validation studies. Patients were classified as responders or non-responders to benznidazole treatment based on their individual PCR results using two different criteria (see below for details). (**B**) Age distribution of patients before and after treatment.

To identify and compare *T*. *cruzi* antigens recognized by patients with Chagas disease before and after treatment, we selected a cohort of 20 patients with variable ethnic origins (but mostly male) with median age of 47 (min. 35, max. 66 years) (**Fig 1A and 1B**), who completed at least 75% of the benznidazole treatment (**Tables 1 and S1**) (19/20 patients completed the full treatment). All patients had chronic disease with varied clinical status at the beginning of the treatment. Before treatment, patients were tested for the presence of the parasite by multiple PCR assays and all patients selected for this study presented at least one positive result at the pre-treatment screening and/or the PCR test performed at baseline. Treatment lasted for 60 days and PCR screenings were performed at baseline (Time 0, **T0**) throughout the first year and then 3 years after the beginning of treatment (year 3, **T3**) with variable results for each patient (**Table 1**). We used data from the multiple PCR tests that were performed during the follow-up period to determine patient’s response to benznidazole: **responder** (PCR negative) or **non-responder** (PCR positive). Because there is not yet a definite guideline of how to assess whether a patient is considered free of disease, we opted to employ two distinct criteria. For the first criterion, if a patient was PCR negative at T3, he/she was considered a responder. According to criterion #1, 16 patients were responders while 4 remained as non-responders (**Table 1**). For the second criterion, if any of the PCR tests were positive during the follow-up period, the patient was considered a non-responder. Applying criterion #2, we observed that 9 patients were responders while 11 remained as non-responders (**Table 1**). Age distribution did not correlate with patients’ response to treatment (**Fig 1B**).

**Table 1:** Clinical characteristics for the cohort of patients.

Patient	Gender	Clinical status	ECG (base)	ECG (3^rd^ year)	PCR result				Treatment outcome	
					60 days	6 months	12 months	3 years	Criterion #1	Criterion #2
1	M	C	Normal	Ventricular repolarization	-	-	-	+	NR	NR
2	M	C	Altered	Right Bundle Branch Block	-	-	+	+	NR	NR
3	M	I	Altered	Left Bundle Branch Block	-	+	-	+	NR	NR
4	F	C	Altered	Right Bundle Branch Block	-	+	-	+	NR	NR
5	M	C	Normal	Ventricular repolarization	-	-	-	-	R	R
6	M	C	Altered	-	-	-	-	-	R	R
7	F	I	Normal	Normal	-	-	-	-	R	R
8	F	I	Altered	-	-	-	-	-	R	R
9	M	Mi	Altered	Normal	-	-	-	-	R	R
10	M	I	Normal	Normal	-	-	-	-	R	R
11	M	I	Normal	Atrioventricular block	-	-	-	-	R	R
12	M	C	Altered	Right Bundle Branch Block	-	-	-	-	R	R
13	M	Mi	Normal	Normal	-	-	-	-	R	R
14	M	C	Normal	Ventricular repolarization	-	-	+	-	R	NR
15	F	C	Normal	Normal	+	-	-	-	R	NR
16	M	I	Normal	-	+	+	-	-	R	NR
17	F	I	Altered	Ventricular repolarization	+	+	-	-	R	NR
18	M	C	Altered	Right Bundle Branch Block	+	-	-	-	R	NR
19	M	C	Altered	Ventricular repolarization	-	+	-	-	R	NR
20	M	I	Normal	Sinus rhythm	+	+	-	-	R	NR

Gender: male (M), female (F). Clinical Status: cardiac (C), indeterminate (I), mixed (Mi) form of the disease. Treatment outcome after 3 years: non-responder (NR) or responder (R). PCR for T. cruzi presence were performed at 60 days, 6 months, 12 months and 3 years after the beginning of treatment. ECG: Electrocardiogram.

### gPhage display screening

**Fig 2:**
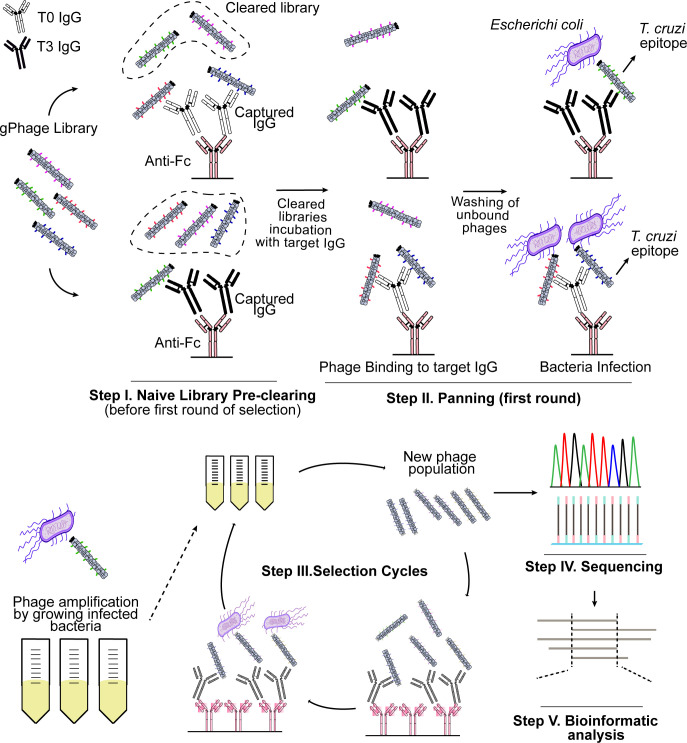
Identification using gPhage of Benznidazole associated-antigen. Scheme illustrating the two-tier gPhage selection protocol.

We used the gPhage technology [13,14] to map the antigen profile of each individual patient before and after treatment. gPhage is a combinatorial technology that allows for the identification of antigens and epitopes recognized by antibodies. This is achieved by fragmenting the whole genome of the parasite and inserting each individual fragment into the filamentous phage genome. The end result is a large collection of phage particles, each displaying a unique peptide encoded by the parasite genome. The gPhage library used in this study had approximate 100-fold coverage of the corresponding *T*. *cruzi* proteome [13]. If an immobilized antibody from a Chagas patient recognizes the antigen/epitope displayed by an individual phage particle, it is then captured and can be recovered and the antigen identified by DNA sequencing (**Fig 2**).

To map the antibody antigen profile of Chagas patients before and after benznidazole treatment, sera from each patient was collected at the beginning of the treatment (T0) or 3 years after baseline (T3). In order to enrich for the identification of antigens specifically recognized by the target antibody (i.e., T0 or T3), a gPhage library pre-clearing step was included; thus, we performed for each individual patient a two-tier panning procedure. For instance, we first pre-cleared the gPhage library using serum at baseline (T0) before performing the phage selection on IgG from serum at year 3 (T3), and vice-versa (**Fig 2**). In all cases, we performed four rounds of selection. We then selected either the 3rd or 4th round (the round with the highest enrichment in phage particles captured by the target IgG in comparison with the 2^nd^ round) for large-scale DNA sequencing in order to identify the antigens and epitopes selected during the biopanning.

### Large-scale DNA sequencing and Bioinformatic analysis

Following the gPhage selection, we used large-scale sequencing [16] and our in-house bioinformatic pipeline to identify antigens and epitopes recognized by IgG from each individual patient, at T0 and T3 [13]. In summary, we observed that on average there was significant phage enrichment for most patients when comparing the round used for sequencing (3^rd^ or 4^th^) with the second round (median 7, min = 0.13 and max = 32) (first round was not quantified to minimize the loss of putative antigens). We obtained on average 228 thousand reads per sample (min. = 107,642, max. = 386,946 reads) (**S2 and S3 Tables**). As we did in our previous study [13,14], all individual reads had to be present at least twice within a given sample. By removing all singletons, we minimized sequencing errors and increased our confidence in the final list of antigens and epitopes used for this study. Insert sequences were also aligned to the *T*. *cruzi* genome and those that did not have at least 95% identity were discarded.

**Fig 3:**
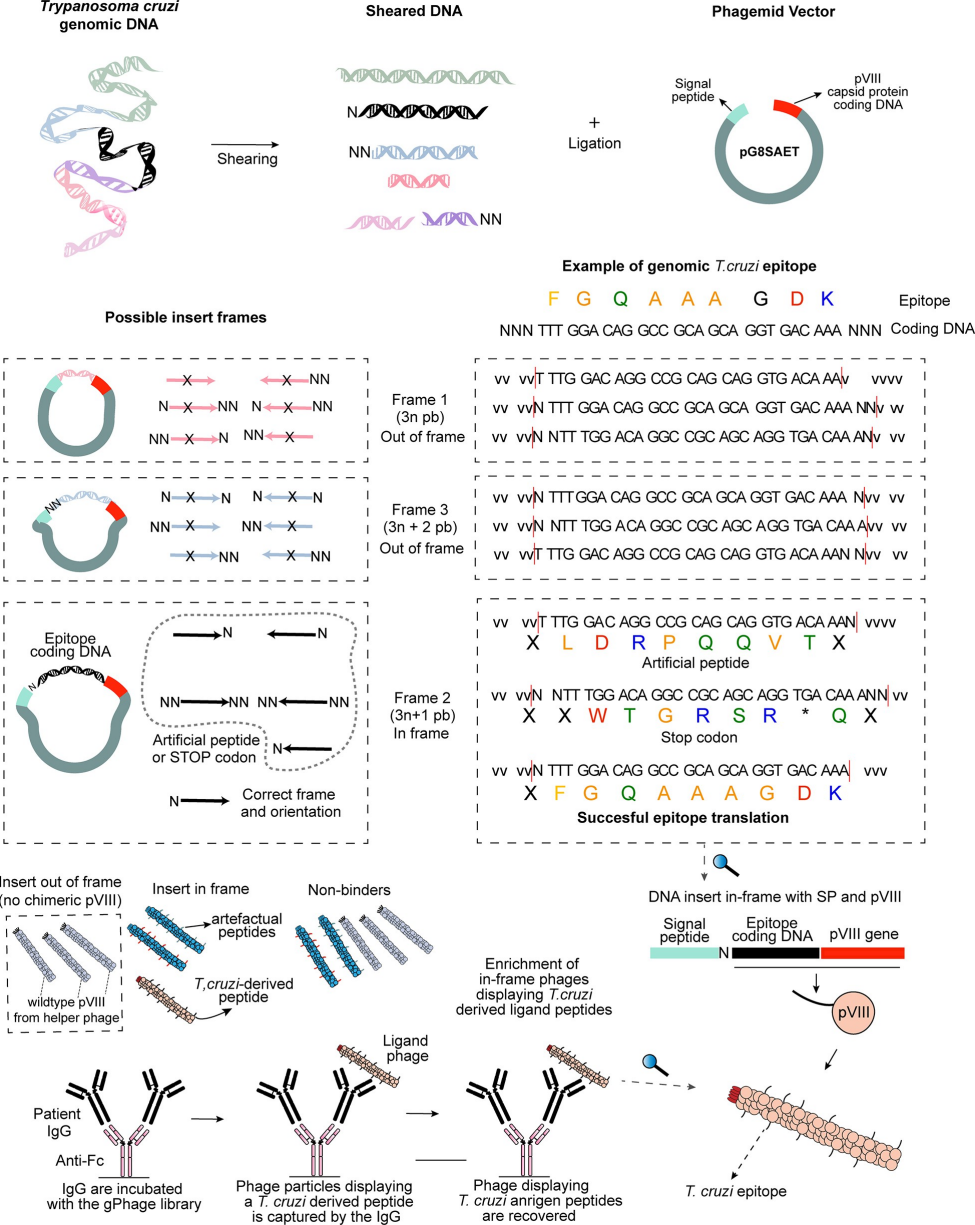
gPhage library construction and phage selection. DNA inserts (fragments of *T*. *cruzi* genomic DNA) were cloned into the gPhage vector (**top**). Because inserts were randomly inserted into the vector into any of all possible reading frames, only a fraction of phage particles displays a *T*. *cruzi*-derived peptide (**middle**). However, with the successive rounds of biopanning, only phage displaying a *T*. *cruzi*-derived peptide are selected and enriched while the remaining phage particles are removed with wash (**bottom**).

Because of the characteristics of the gPhage library (phagemid), not all phage particles display an exogenous peptide. This is because during library construction, inserts are randomly inserted into the phage genome, and not all are in the correct reading-frame to produce a real *T*. *cruzi* epitope fused to the pVIII chimeric protein (**Fig 3**). In order to be effectively displayed in the bacteriophage surface, the insert has to be in frame with the vector’s signal peptide sequence (5’) and the bacteriophage gene VIII (3’) (**Fig 3**). But it is also possible for a *T*. *cruzi* DNA insert cloned in the wrong reading-frame to have an open-reading frame (ORF) that results in the display of an artefactual non-*T*. *cruzi* peptide (**Fig 3**). These inserts were mostly removed by aligning the encoded peptide against the *T*. *cruzi* proteome.

Because our initial gPhage library was constructed by randomly cloning fragments of the parasite’s genome, it contained a modest 8.6% of phage particles displaying a *T*. *cruzi* antigen (nevertheless, the coverage of our gPhage library is over 100-times the parasite’s encoded-proteome). Hence, upon selection, we observed an enrichment in the number of phage particle displaying a *T*. *cruzi* antigen: on average, 51% for both patients in T0 and T3 (min = 0, max = 100%) (**Fig 3** and **S2 and S3 Tables**). This indicates that the gPhage selection was successful: phage particles that did not carry a *T*. *cruzi* antigen were washed away during the selection process, while those that contained and epitope recognized by the patient’s IgG were captured and enriched during the successive rounds of selection (**Figs 2 and 3**). However, because of our two-tier selection process, enrichments were lower than we have observed previously [13]. On the other hand, this decrease was expected, considering that we performed a pre-clearing step using sera from a Chagas patient that clearly reduced the number of antigens recovered in the second step of the selection.

**Fig 4:**
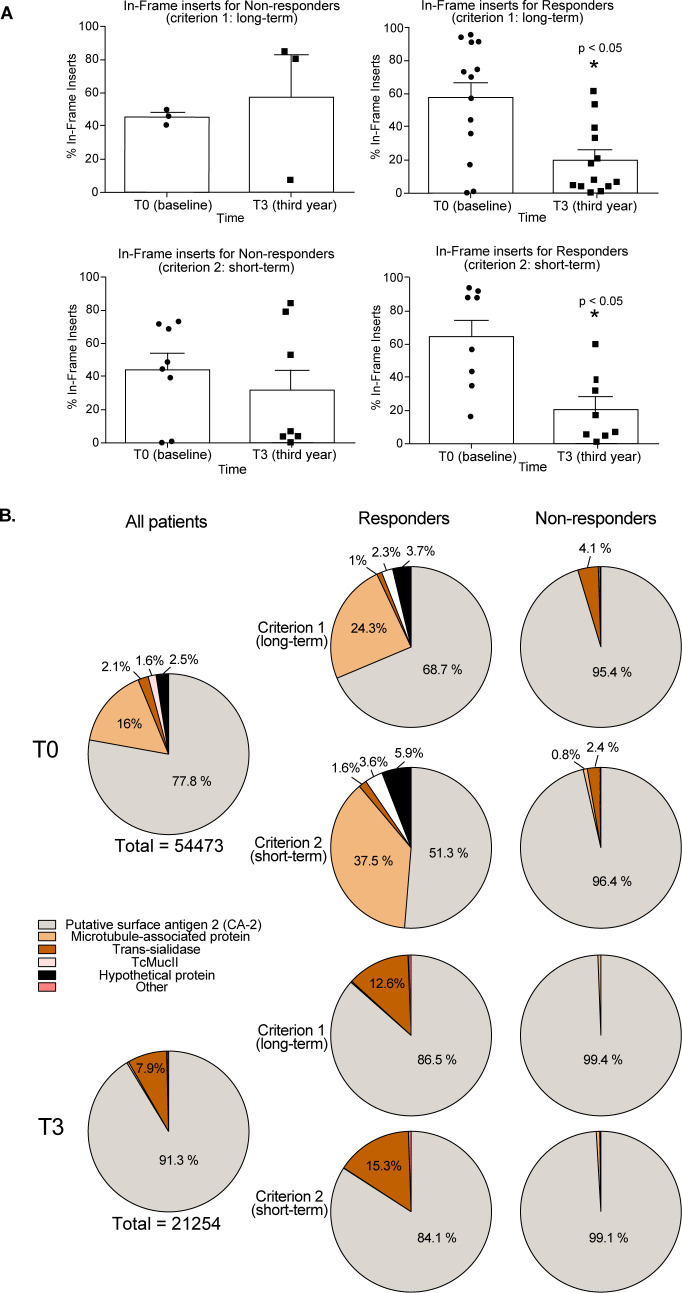
gPhage predicts treatment outcome. (**A**) Bar graphs displaying the percentage of in-frame inserts for responders and non-responders according to both short- and long-term criteria. Responders showed a significant reduction in the percentage of in-frame inserts at T3 compared with T0 (Student T-test). (**B**) Pie graphs summarizing antigens recognized by responder and non-responder patients at T0 and T3 according to each criterion.

After our bioinformatic pipeline processing was complete, we used the enrichment data as proxies for patient sera immunogenicity. For both criteria, we observed a positive correlation between the percentage of in-frame inserts and the PCR status of the patients. Patients that were classified as responders based on their PCR results according to both criteria showed a significant (*p* = 0.0081, criterion #1 or *p* = 0.0246, criterion #2) lower percentage of in-frame inserts recovered by T3 IgG (mean = 16.2, criterion #1 and mean = 23.1, criterion #2) in comparison with T0 (mean = 45.9 for criterion #1 or mean = 57.5 for criterion #2) (**Fig 4A**), which may be related to a sera decrease of *T*. *cruzi-*specific antibodies. No significant difference (*p* > 0.05) was observed for non-responder patients when comparing T3 (mean = 42.9, criterion #1 and mean = 23.1, criterion #2) with T0 (mean = 33.7, criterion #1 and mean = 32.0, criterion #2) (**Fig 4A**). These data indicate that gPhage may be useful to predict the outcome of treatment and that antigens captured during our biopanning procedure may reflect patient’s disease status.

### Antigen profile before and after benznidazole treatment

Given the observed correlation between our gPhage selection and the PCR status of patients, we next compared the antigen profile of responders and non-responders, before and after treatment. Because there was no significant enrichment of in-frame gPhage particles displaying a *T*. *cruzi* antigen in comparison with the naïve gPhage library for some of the patients, we only considered data from patients with a positive selection of at least twice (17.2%) the value of in-frame inserts present in the initial naïve gPhage library (8.6%). So, for these analyses we had data from 13 patients at T0 and from 7 patients at T3.

Applying our bioinformatic pipeline [13], we aligned the translated inserts to the *T*. *cruzi* proteome and considered only sequences with at least 60% of identity (to allow for antigen variability due to different strains and the numerous multigene families). This resulted in 75,727 *T*. *cruzi*-derived peptides that were recognized by all patients (a total of 54,473 peptides at T0 and 21,254 peptides at T3). However, many peptides represent distinct fragments from the same antigens. Thus, we next performed a clusterization step to generate a list of non-redundant antigens and epitopes, as previously described [13,14]. This resulted in 80 clusters of antigens (59 at T0 and 21 at T3) (**S1 Data**). Here, it is interesting to note the reduction (from 59 to 21, or 64%) in the number of antigens/epitopes recognized by patients after 3 years of treatment with benznidazole. These data agree with the reduction in the percentage of in-frame inserts (**Fig 4A and S2 and S3 Tables**) and previous reports of seronegativation following benznidazole treatment [20–22].

We observed that for responders and non-responders, the most predominant antigenic cluster recognized by patients was the CA-2 surface protein (B13 epitope), with 24,537 peptides forming 17 clusters at T0 (4 for non-responders and 13 for responders) and 19,407 peptides constituting 12 clusters at T3 (3 for non-responders and 9 for responders) (**Tables 2 and 3 and Fig 4B**). Another well-represented antigen was the microtubule-associated protein (MAP), mainly recognized by patients within the responder group at T0, with 8,698 peptides forming 12 clusters for responder patients at T0. Only two patients (#10 and #11) showed non-CA2 antigens as immunodominant and predominant clusters: Mucin (TcMucII) and MAP, respectively. Although highly immunogenic and the largest multigene family, members of the trans-sialidase were not among the most abundant antigens/epitopes identified, with the notable exception of one patient (#12 at T3) who had a trans-sialidase antigen as the immunodominant epitope. Interestingly, this particular trans-sialidase epitope is a long-tandem repeat (with over 50 repetitions of the identified epitope—protein ID# PBJ71599.1), a common element in our gPhage screening. Finally, the R27-2 antigen, another long-tandem repeat antigen that shares similarity with the CRA1/2 antigens [23], and several hypothetical proteins were also recognized by IgG of multiple patients, mostly at T3. In sum, our data are in agreement with previous observations that benznidazole treatment has a significant effect on the patient’s antibody response, reducing anti-*T cruzi* antibodies although recognized antigens appear to vary between individuals, with no recognizable signature antigens for responders and non-responders.

**Table 2:** Antigens and epitopes preferentially recognized by patients’ IgG at the beginning of treatment (T0).

Patient	Antigen	Accession	Frequency (%)	Consensus sequence
1	<17.2% in-frame inserts			
2	Putative surface antigen 2 (CA-2) Trans-sialidase, putative, partial Trans-sialidase, putative, partial Putative trans-sialidase Trans-sialidase, putative	PWU84979.1 EKF99511.1 EKF98285.1 PWU90932.1 EKG00226.1	95.69 3.73 0.40 0.13 0.05	GQAAAGDKPSPFGQAAAGDKPPPFGQAAAGDKPSPFGQAAAGDK KASVHVDGESLGNEEVPLTGEKPPE TSLGEEEVPLTGEAPLGLV PDSFSSTNVSGGVDAAPAPSSTASG APSSTASGETKIPSELNATVPSDHDILLEFRELA
3	Putative surface antigen 2 (CA-2) Putative surface antigen 2 (CA-2)	PWU84979.1 PWU84979.1	99.69 0.31	QAAAGDKPPPFGQAAAGDKPSPFEQAAAG GDRPSPFEQAAAGAKPSPFGQ
4	Putative surface antigen 2 (CA-2) Mucin TcMUCII Mucin TcMUCII, putative Hypothetical protein TCSYLVIO_010170 Monoglyceride lipase, putative, partial Hypothetical protein TCSYLVIO_009815 Hypothetical protein MOQ_008444, partial Putative 60S ribosomal protein Mucin TcMUCII, putative Hypothetical protein	PWU84979.1 XP_808990.1 EKF29049.1 EKF98926.1 EKF27476.1 EKF99266.1 EKF27823.1 PWU84687.1 EKF29049.1 XP_822001.1	40.49 23.93 9.82 9.82 4.91 3.68 2.45 1.84 1.84 1.23	AAGDKPPPFGQAAAGDKPSPFGQAAAG PSTTTTEAPTTTTTRAPSRLREID NTARNTEAPITTTTTRAPSRFREID HDIYHQRNPNSSGSVGRRGGVWGRTVSPASTEQGL VPLTARYGAEMMRAIDD ETVDALNEKVWTAEFRQIDTE TDTTPLVIVGPETSVAPVAAQRAIDTV VTGTAMPRGMQAFPLRDSAETI AGNTEAPATATTRAPSRLREID RQRLVDPSEPPTSPASTEMDETGKAST
5	Putative surface antigen 2 (CA-2) Putative microtubule-associated protein Hypothetical protein C4B63_133g26 Microtubule-associated protein, partial Hypothetical protein C4B63_76g64 Hypothetical protein C4B63_76g64 Surface antigen 2 (CA-2) Hypothetical protein Putative surface antigen 2 (CA-2) Putative microtubule-associated protein	PWU84979.1 PWU97874.1 PWU86111.1 XP_803031.1 PWU88332.1 PWU88332.1 XP_818927.1 XP_813515.1 PWU84979.1 PWU97874.1	70.41 24.05 4.87 0.32 0.20 0.06 0.04 0.02 0.02 0.02	GQAAAGDKPSPFGQAAAGDKPSPFGQAAAGDKPSPFGQAAAGDK VDPSAYKRALPQEEEEDVGPRHVDPDHFRSTTQ PFKSVFGAPSSTDAKPPAESPFKS MGPSAQNYDTQEEEDVGPRHVDPDHFRST ATHERAVEALAAEEDAARGQLVGGE AAVDELGEAFRSATHERAVEALAAEEDA SPFGQAAAGDKPPLFGQGTVFDAS DAKPPAESPFKGGFGAPSSTVAKPPGESPFKN AAGDKPPPVEQAAGGDRPSPFEQA YKRALPEEGQGDLGPRQVDPDHFRSTTQDA
6	putative surface antigen 2 (CA-2) hypothetical protein C4B63_9g493 putative mucin TcMUCII putative microtubule-associated protein hypothetical protein C4B63_9g493 mucin TcMUCII, putative	PWU84979.1 PWU99324.1 PWU85205.1 PWU97874.1 PWU99324.1 EKF29049.1	76.33 21.11 2.17 0.28 0.06 0.04	SPFGQAAAGDKPPPFGQAAAGDKPSPFGQAAAGDKPPL SAPAAGGFGSATTTSAPAVGGFGSATT TPSTTTTGTPTTTTTRAPSRLREIDS LPQEEQEDVGPRHVDPDHFRSTTQDAY VGGFGSAAHTSTPGVGGFGSATTT PINTAGKTEAPTTTTITHAPSRLREID
7	Too few peptide sequences			
8	Putative surface antigen 2 (CA-2)	PWU84979.1	100.00	DKPPPFGQAAAGDKPSPFGQAAAGDKPSPF
9	<17.2% in-frame inserts			
10	Mucin TcMUCII Mucin TcMUCII Mucin TcMUCII, putative Mucin TcMUCII, putative, partial	XP_821913.1 XP_820005.1 EKG05373.1 EKG03739.1	95.90 2.97 0.85 0.28	TEASTTTTTRAPSRLREID TTTTTTEAPNTTIPRAPSRLREID TTTTTTADPTTTSARTPSRLREID TTTTTTSAPEAPSNTTMNTEAPTPTTSRAPLRLREIDV
11	Putative microtubule-associated protein Putative surface antigen 2 (CA-2) Flagellar attachment zone protein 1 Putative microtubule-associated protein	PWU97874.1 PWU84979.1 PWU95940.1 PWU97874.1	93.43 6.45 0.10 0.01	ALPQEEEEDVGPRHVDPDHFRSTTQ QAAAGDKPPPFGQAAAGDKPSPFGQAAAGDKPS EELEQKAAENERLAEELEQKAAENE EEQEDVGPRHVGPDQFPPTTQDAYRPVDPS
12	Putative surface antigen 2 (CA-2) Putative trans-sialidase Putative microtubule-associated protein Putative surface antigen 2 (CA-2)	PWU84979.1 PWU87189.1 PWU97874.1 PWU84979.1	71.29 28.31 0.24 0.16	AAGDKPSPFGQAAAGDKPSPFGQAAAGDKPLPFEQA MPAGTSEEGSRDDSPMPAGASEEGSRDD SAYKRALPQEEEEDVGPRHVDPDHFRST AAGEKLPFGKAAAGDKPPPFGQA
13	Putative surface antigen 2 (CA-2) Putative microtubule-associated protein Hypothetical protein Hypothetical protein	PWU84979.1 PWU97874.1 XP_820768.1 XP_810212.1	96.10 1.74 1.67 0.49	SPFGQAAAGDKPSPFGQAAAGDKPSPFGQA LPQEEQEDVGPRHVDPDHFRSTTQ DSVTLTSLWSSRTAQARPASMRLVDGV PAVGGFGSATTTSAPAAGGFGSATTTSAPA
14	Putative surface antigen 2 (CA-2) Putative surface antigen 2 (CA-2)	PWU84979.1 PWU84979.1	96.84 3.16	GQAAAGDKPPPFGQAAAGDKPSPFGQ DKPSPFGQVAAGEKPPPFGQAAAGEK
15	<17.2% in-frame inserts			
16	Putative surface antigen 2 (CA-2) Retrotransposon hot spot (RHS) protein	PWU84979.1 EKF99800.1	99.95 0.05	GQAAAGDKPPPFGQAAAGDKPSPFGQA MNCTPCGPFCWGMAGGYVGWNYCLRQHGRRLTFM
17	Putative surface antigen 2 (CA-2) Putative microtubule-associated protein Putative microtubule-associated protein Putative microtubule-associated protein Putative microtubule-associated protein	PWU84979.1 PWU97874.1 PWU97874.1 PWU97874.1 PWU97874.1	97.31 2.66 0.01 0.01 0.01	AAGDKPPPFGQAAAGDKPPPFGQAAAGDKP ALPQEEEEDVGPRHVDPDHFRSTTQD SAYKRALPQEEEGGVGPGPVDPAHFRSP SAYKRALPQEEEEDVGPGQVDPDQFRWT SAYKRALPQEKEGDGGPGHVVPDHFRST
18	<17.2% in-frame inserts			
19	<17.2% in-frame inserts			
20	<17.2% in-frame inserts			

**Table 3:** Antigens and epitopes preferentially recognized by patients’ IgG at the end of treatment (T3).

Patient	Antigen	Accession	Frequency (%)	Consensus sequence
1	<17.2% in-frame inserts			
2	Putative surface antigen 2 (CA-2)	PWU84979.1	100.00	FGQAAAGDKPPPFGQAAAGDKPSPFGQ
3	Putative surface antigen 2 (CA-2) Putative microtubule-associated protein Putative surface antigen 2 (CA-2) Putative trans-sialidase	PWU84979.1 PWU97874.1 PWU84979.1 PWU87189.1	60.12 27.98 11.31 0.60	AAGDKPPPFGQAAAGEKPSP VDPDHFRSTTQDAYRPVDPSAYK FGQAAAGDKPPPFGQA MPAGTSEEGSRDDSSMPAGT
4	<17.2% in-frame inserts			
5	<17.2% in-frame inserts			
6	<17.2% in-frame inserts			
7	<17.2% in-frame inserts			
8	<17.2% in-frame inserts			
9	<17.2% in-frame inserts			
10	<17.2% in-frame inserts			
11	Putative surface antigen 2 (CA-2) Putative microtubule-associated protein Putative surface antigen 2 (CA-2) Putative surface antigen 2 (CA-2)	PWU84979.1 PWU97874.1 PWU84979.1 PWU84979.1	94.25 4.60 0.57 0.57	SPFGQAAAGDKPPPFGQAAAGDKPSPFG ALPQEEEEDVGPRHVDPDHFRSTTQDA DKPSLFGQAAAGDNPSPFGQAAAGDK DKPSPFGQAAGGEKPPPFGPAAAGDK
12	Putative trans-sialidase Putative surface antigen 2 (CA-2) R27-2 protein Putative trans-sialidase	PWU87189.1 PWU84979.1 XP_818212.1 PWU87189.1	89.89 6.84 3.16 0.11	MPAGTSEEGSRGDNSMPAGASEEGSRG DKPPPFGQAAAGDKPSLFGQAAAGDKP KVAEAEKQRAAEATKVAEAEKQR SSMPAGTSQEGIRDDRSMRGGASEEGSRDD
13	Putative surface antigen 2 (CA-2) Hypothetical protein C4B63_22g72 Putative surface antigen 2 (CA-2)	PWU84979.1 PWU95420.1 PWU84979.1	99.97 0.02 0.01	SPFGQAAAGDKPPPFGQAAAGDKPSPFGQAAAG QEGQAETKRNSVRRGDDPPSAAATPAGTT DKPPPFGQAASGEKPSPFGKAAAGEKPSPF
14	<17.2% in-frame inserts			
15	<17.2% in-frame inserts			
16	<17.2% in-frame inserts			
17	Putative surface antigen 2 (CA-2) Putative microtubule-associated protein Hypothetical protein	PWU84979.1 PWU97874.1 XP_803285.1	97.78 1.14 1.09	AAGDKPSPFGQAAAGDKPPPFGQAAAGDKP QEDVGPRHVDPDHFRSTTQD SRHWWQCHEWRGTGRAQGLGVEMLVKAKDGGSEPLT
18	<17.2% in-frame inserts			
19	Putative surface antigen 2 (CA-2) Putative surface antigen 2 (CA-2)	PWU84979.1 PWU84979.1	93.81 6.19	AAAGDKPSPFGQAAAGDKPSPFG PPFGQAAADDKPPPFGQAAAGEK
20	<17.2% in-frame inserts			

### Epitope validation

**Fig 5:**
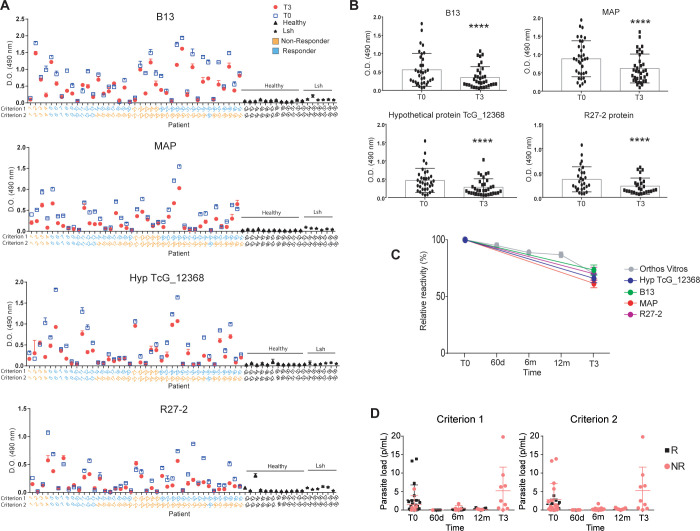
Antigen validation. (**A** and **B**) ELISA reactivity against select antigens for sera (T0 and T3) of patients classified as responders (**blue**) or non-responders (**orange**) according to each criterion. Sera from individual patients (dilution 1/200) (including 21 additional patients not used for the gPhage selection) were tested against synthetic peptides encoding each individual antigen CA-2 (B13), Microtubule-associated protein (MAP), Hypothetical protein TcG_12368 and R27-2 (peptide 4) (Wilcoxon paired-rank test: **** p<0.0001). (**C**) Reactivity of sera from individual peptides in comparison with OV assay. (**D**) Parasite load (quantified by PCR) for the cohort of 41 patients. OV: Orthos Vitros serology assay. R: responders. NR: non-responders. (Lsh = sera from patients with Leishmaniosis).

Based on the antigen profile we obtained, we next tested by ELISA (**Fig 5**) the reactivity of sera from patients who were classified as responders and non-responders. For that end, sera collected at baseline (T0) and T3, including an additional cohort of 21 new patients treated with benznidazole but for which we have no gPhage data (**S1 Table**), were tested against the two immunodominant antigens identified in our study, CA-2 (B13) and MAP. We also include two minor epitopes, the R27-2 and the hypothetical protein TcG_12368. Synthetic peptides encoding each epitope were immobilized on microtiter wells and reactivity of sera from each patient (T0 and T3) was quantified by ELISA. We observed that, overall, sera from all patients reacted with at least one of the antigens and that reactivity was consistently lower with sera collected at T3 than with sera collected at the beginning of treatment (T0) (**Figs 5A, 5B and S1**). These results agree with previous studies that indicate a decrease in anti-*T*. *cruzi* antibodies in plasma upon benznidazole treatment [21,22] and with our own gPhage results. All antigens showed null or very low reactivity towards the control samples (blood bank donors that tested negative for Chagas disease) or patients with Leishmaniasis, indicating that the markers identified by gPhage were specific for Chagas disease (**Fig 5A**). A similar profile was observed for B13, MAP, R27-2 and Hypothetical protein TcG_12368. Next, we compared our ELISA results with data obtained using the Orthos Vitros (OV) commercial kit, which showed a similar decrease in antibody reactivity (**Fig 5B and 5C**), with a reduction of reactivity not necessarily correlated with the parasite relapse in non-responder patients (**Fig 5D**). However, although there was a similar reduction in the reactivity towards *T*. *cruzi* antigens in sera collected at T3 and T0 among responder and non-responders (quantified by the ratio T0/T3), data from ELISA and OV could not predict patient outcome with regard to benznidazole treatment regardless of the criteria used to assess patient response to treatment (**Figs 5B, 5C and S1**). The decrease in reactivity at T3 in comparison with T0 was 73.5% for B13, 61.7% for MAP, 66.1% for Hyp TcG_12368, and 70.1% for R27-2. It is interesting that both serological assays were not able to predict patient outcome (clearance in parasitemia) as gPhage did, although both methods correctly predicted a reduction of parasite load induced by benznidazole treatment based on a reduction in anti-*T*. *cruzi* antibodies (**Fig 5D**).

### Conclusions and limitations of the study

In this work, we studied the landscape of the antibody response of individual patients with Chagas disease following a full course treatment with benznidazole. We show that gPhage technology [13] can be utilized to screen and quantify the antibody response of Chagas disease patients and, in agreement with previous reports [20–22], we observed that benznidazole treatment led to a significant reduction of anti-*T*. *cruzi* antibodies and the overall number of epitopes/antigens recognized by sera IgG. Our study also highlights the diagnostic value of previously identified antigens/epitopes (CA-2 and MAP) as possible markers for cure. Interestingly, however, the in-frame data from gPhage could be used as proxies to predict treatment outcome, a result that neither ELISA nor the OV data could achieve using samples from the same cohort of patients. One possible explanation for this is that gPhage relies on an unbiased library containing all (or most of the) antigens encoded in the parasite’s genome to probe each patient individually. In that sense, gPhage is similar to a precision medicine diagnostic tool, as opposed to serological assays that rely on a single or a combination of pre-determined antigens, some of which not all patients might react to.

Limitations of the present study are the relatively small cohort of patients and the lack of specific guidelines for determining whether a patient is a responder or a non-responder to benznidazole treatment. Hence, we proposed and evaluated two criteria to assess patient response to treatment, which we defined as long- and short-term criteria. Most importantly, regardless of the criterion used, the gPhage data could be used to predict treatment outcome. Therefore, the use of two criteria mitigated the limitation of our small cohort.

Another aspect that merits specific comments is the strategy to perform the gPhage selection using a pre-clearing step. By removing from the gPhage library antigens that reacted with anti-*T*. *cruzi* antibodies found in a given serum sample (i.e., before treatment) we favored the selection of antigens specific for antibodies found in the next paired serum sample (i.e, after treatment). However, this strategy seems to have reduced significantly the number of antigens mapped, including samples for which we recoved very few phage particles and, therefore, could not identify any preferentially selected antigen or epitope. On the other hand, this strategy may have allowed for a more direct comparison (quantification) of gPhage data (before/after treatment) for each individual patient. But because it limited the number of antigens identified, one should be cautious when analyzing the somewhat small list of antigens in our study. A more complete list of antigens and epitopes recognized by patients in each stage of Chagas disease can be found in our original study [13].

Although the gPhage selection strategy seems to have favored the selection of major immunodominant epitopes, such as CA-2, Mucin and MAP, we did observe an intriguing trend. Overall, sera obtained from patients before treatment reacted with a larger diversity of antigens (**Fig 4B**); after benznidazole treatment, the great majority of patients reacted preferentially with the CA-2 antigen, including one patient that switched from reactivity with the MAP antigen to CA-2 (patient #11; **Tables 2 and 3**). Unfortunately, the small number of patients in the cohort limited further association analyses between specific antigens or epitopes versus response to treatment, disease status or clinical evolution. Future studies performed with a larger cohort of patients, with and without the pre-clearing step to favor the identification of minor antigens/epitopes as well, will be necessary to further evaluate the power of gPhage as a diagnostic tool. In sum, gPhage may prove to be an important differential for developing new diagnostic tools and for the discovery of novel markers relevant for cure of Chagas disease.

## Supporting information

S1 Table Complete clinical and diagnostic data for patients used in phage display panning. (XLSX)

S2 Table Summary of bioinformatic analyses for patients at T0. (XLSX)

S3 Table Summary of bioinformatic analyses for patients at T3. (XLSX)

S4 Table Summary of antigenic clusters for selected peptides. (XLSX)

S1 Data Clusters of antigens for individual patients. (RAR)

S1 Fig Antigen validation (individual patients) and Orthos Vitros assay. (PDF)
