# Supplementary material for: Anti-Trypanosoma cruzi antibody profiling in patients with Chagas disease treated with benznidazole assessed by genome phage display
Source: PLoS Negl Trop Dis. 2023 Jan 6;17(1):e0011019. doi: 10.1371/journal.pntd.0011019 (PMC9851536; doi:10.1371/journal.pntd.0011019)
Supplement: S1 Fig — (PDF) [file pntd.0011019.s006.pdf]

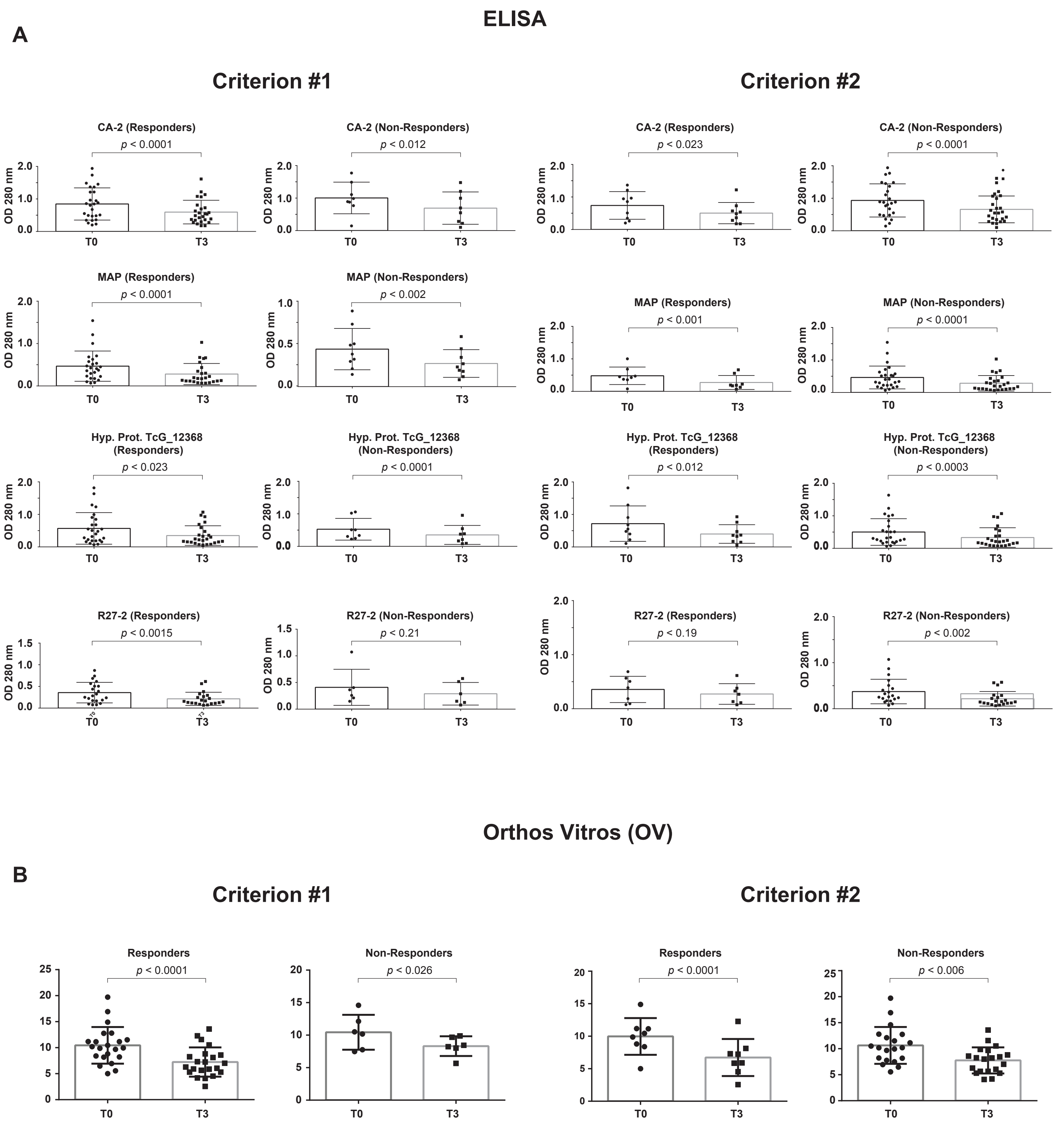

Supplementary Figure S1. *Antigen validation (individual patients) and Orthos Vitros assay.* (**A**) ELISA reactivity against select antigens for sera (T0 and T3) of patients classified as responders or non-responders according to each criteria. Sera from individual patients (dilution 1/200) (including 21 additional patients not used for the gPhag e selection) were tested against synthetic peptides encoding each individual antigen CA-2 (B13), Microtubule-associated protein (MAP), Hypothetical protein TcG_12368 and R27-2 (peptide 4). Patients that showed reactivity below the cut-off value were not included. (**B**) Reactivity of sera from individual peptides using the Ortho Vitros (OV) assay. Statistical analyes: Wilcoxon paired-rank test.
